# Illusionary Self-Motion Perception in Zebrafish

**DOI:** 10.1371/journal.pone.0006550

**Published:** 2009-08-12

**Authors:** Ying-Yu Huang, Markus Tschopp, Stephan C. F. Neuhauss

**Affiliations:** 1 Department of Biology, Swiss Federal Institute of Technology (ETH) Zurich, Zurich, Switzerland; 2 Brain Research Institute of the University of Zurich, Zurich, Switzerland; 3 Institute of Zoology, University of Zurich, Zurich, Switzerland; University of Maryland, United States of America

## Abstract

Zebrafish mutant *belladonna* (*bel*) carries a mutation in the *lhx2* gene (encoding a Lim domain homeobox transcription factor) that results in a defect in retinotectal axon pathfinding, which can lead to uncrossed optic nerves failing to form an optic chiasm. Here, we report on a novel swimming behavior of the *bel* mutants, best described as looping. Together with two previously reported oculomotor instabilities that have been related to achiasmatic *bel* mutants, reversed optokinetic response (OKR) and congenital nystagmus (CN, involuntary conjugate oscillations of both eyes), looping opens a door to study the influence of visual input and eye movements on postural balance. Our result shows that looping correlates perfectly with reversed OKR and CN and is vision-dependent and contrast sensitive. CN precedes looping and the direction of the CN slow phase is predictive of the looping direction, but is absent during looping. Therefore, looping may be triggered by CN in *bel*. Moreover, looping in wild-type fish can also be evoked by whole-field motion, suggesting that looping in a *bel* mutant larvae is a result of self-motion perception. In contrary to previous hypotheses, our findings indicate that postural control in vertebrates relies on both direct visual input (afference signal) and eye-movement-related signals (efference copy or reafference signal).

## Introduction

Zebrafish mutant *belladonna* (*bel*) was originally isolated in a large screen of mutations affecting retinotectal axon pathfinding [1]. In a subsequent visual behavioral screen using optokinetic response (OKR), *bel* mutants were found to often display a curious reversal of eye movement triggered by motion in the surround [2]. Genetic mapping revealed that the *bel* mutant is a result of mutations in the Lhx2 gene (encoding a Lim domain homeobox transcription factor) required for forebrain patterning and midline axon guidance [3]. Thus the *lhx2* (*bel*) mutation causes about 20 to 50 percent of the homozygous *bel* larvae to suffer from a failure of the retinal ganglion cell (RGC) axons to cross the midline, a condition called achiasmia. In contrast to mutants with normal RGC projection (*bel fwd*), achiasmatic larvae (*bel rev*) display two characteristic oculomotor instabilities that can be completely attributed to the RGC misrouting due to a sign-reversed afference signal: reversed OKR and involuntary oscillatory eye movements (congenital nystagmus, CN) [4], [5]. The strong resemblance of the waveform characteristics and behavioral symptoms between CN in *bel rev* and CN in human patients with likely similar underlying neuronal deficits (i.e., optic nerve projection defects) suggests that *belladonna* may be a disease model for axonal misrouting-related CN in humans [5]. Analogous to achiasmatic *bel*, visual pathway abnormalities in humans such as in albinism and non-decussating retinal fugal fiber syndrome (achiasmia) have been consistently associated with CN [6]–[9].

**Figure 1:**
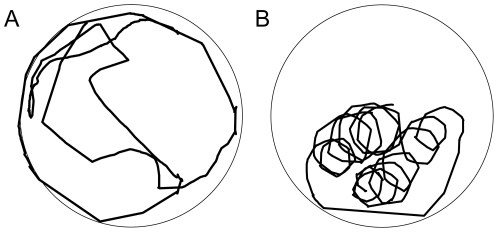
Swimming traces of wt and *bel rev* larvae in a 22-mm-diameter dish exposed to a projected still grating (spatial frequency = 0.06 cycles/deg, contrast = 100%). A, wt swims in random direction or stays still near the dish edge (recording duration = 236 s). B, *bel rev* displays the characteristic looping behavior (recording duration = 55 s).

In this study, we investigated the role of vision in an intriguing swimming behavior, best described as looping, which we have observed in some of the zebrafish *bel* mutants (Figure 1). Instead of swimming in random direction like wild-type (wt) fish, some *bel* mutants constantly swim in circles of varying center and radius. Taking advantage of the evident etiology of CN in the *bel rev* mutant and the repetitive and robust measurement of the zebrafish OKR behavioral assay (see review [10]), we aimed to understand how visual-postural control integrates information from the visual system by investigating the potential causal relationship between the oculomotor (i.e., CN) and postural instabilities (i.e., looping) in the achiasmatic *bel* (*bel rev*) mutants. In particular, we wanted to shed light on whether visual-postural control relies on retinal slip (afference signal), eye-movement-related signals (efference copy or reafference signal), or a combination of the two.

Many people may have experienced a sudden lost of body balance induced by a moving visual background, for example when visiting an IMAX® movie theater. The classic way of thinking about visual postural control is that the afferent signal (retinal slip; i.e., velocity of the image on the retina) serves as the primary input of the visual system to postural control. A number of studies support such a view: For example, body sway is considerably reduced in light compared to darkness, suggesting that vision significantly helps stabilizing posture [11], [12]. Moreover, a moving scene induces the body to incline in the same direction as the scene motion [13].

Conversely, eye movement information may directly influence visual-postural control via either efference copy of motor command or reafference signal from the eye muscles. Recently, it has been proposed that such eye-movement-related signals rather than retinal slip (afference signal) may be key to controlling postural balance [14]–[16]. Both eye movements (spontaneous nystagmus) and body sway in patients with vestibular neuritis were reduced by wearing a fixed-head target to prevent the influence of afferent visual cues [14]. Additionally, pursuing a slowly moving target in darkness induces more body sway than fixating a stable target even though retinal slip is similarly low in both cases [16]. Although strongly suggestive, these studies fail to rule out the possible influence of retinal slip on postural balance. In fact, both the afferent and efferent signal may work together to stabilize posture as has been suggested elsewhere [17].

The two congenital oculomotor instabilities (reversed OKR and CN) in the achiasmatic *bel* mutant provided us with a unique chance to investigate the influence of visual input and eye movements on postural balance. The goal of the present study therefore was to understand how visual input (afferent signal, i.e., retinal slip) or eye-movement-related signals (efference copy and/or reafference signal) influence postural balance by examining the interaction between CN and looping in achiasmatic *bel* (*bel rev*) mutants.

## Results

### Body looping in *belladonna* is correlated with oculomotor instabilities

Instead of swimming in random directions like wild-type (wt) fish, homozygous *belladonna* (*bel*) mutants displayed an intriguing looping behavior that was observed as early as 5 days post fertilization (dpf) and may be retained to adulthood (Figure 1). Looping is distinctive from certain spinning behaviors that are often seen in mutants with a vestibular dysfunction, termed circler mutants [18]. The novel behavior described here would be more aptly termed circling, in the sense that the movement is around an axis centered outside of the larva's body, while circler mutants spin around their body center. We named the behavior “looping” to avoid confusing terminology. Circler mutants turn several times around their longitudinal or vertical body axis and lie on the side afterward. However, the looping of *bel* is a clearly different behavior. Instead of turning around their body axis, they swim in circles. As shown in Figure 1, the center and the radius of the circles can vary in a single subject. Looping can be observed in both clockwise and counterclockwise direction (see Movie S1).

As looping can be interpreted as a deficiency in body balance, the *lhx2* gene mutation could cause looping by affecting the vestibular system. Thus, if looping was due to balancing problems caused by the *bel* (*lhx2*) mutation, most likely all homozygous *bel* mutants regardless of how the optic nerves project (i.e., both *bel fwd* and *bel rev*, see [5]) would be expected to have had the same tendency for such looping behaviors. Alternatively, looping could be a result of the *lhx2*-mutation-related ipsilateral projection of the optic nerves, which has been shown to cause two specific oculomotor instabilities: reversed optokinetic response (OKR) and congenital nystagmus (CN) [4], [5]. Since only about half of homozygous *bel* larvae are achiasmatic and show CN (*bel rev*, since they show a reversed OKR), while the other half form a normal optic chiasm and show normal eye movements without CN (*bel fwd*, for forward (normal) OKR) [4], [5], we can hence distinguish between these hypotheses by asking first if achiasmia-related oculomotor instabilities and looping are correlated. Here we examined the occurrence of both reversed OKR and CN in single isolated looping larvae and the result revealed that all looping *bel* mutants also exhibited reversed OKR (100%, N = 36) and CN (100%, N = 18), implying that only achiasmatic mutants (i.e. *bel rev*) display the looping behavior. Therefore looping is not caused by vestibular defects, but may be related to the oculomotor instabilities in *bel rev*.

### Looping is visual input and contrast dependent and may be triggered by CN

**Figure 2:**
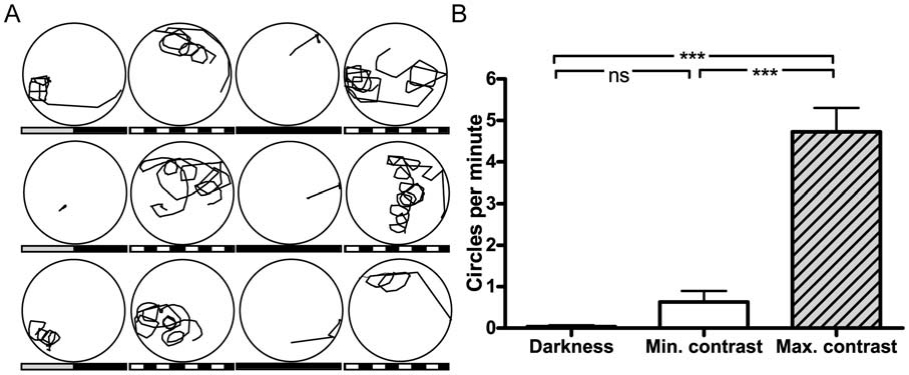
Contrast and visual input dependence of looping. The panels show the swimming traces of a single *bel rev* larva in a 22-mm-diameter dish across 12 subsequent (from a to l) visual stimulus conditions (each panel corresponds to one condition with 60 s recording duration). A, Only few circles were observed when *bel rev* was exposed to a projected gray uniform background (minimal contrast, gray-black bar). A projected black and white grating consistently evoked strong looping (maximum contrast, black-and-white bar). Swimming activity almost completely ceased in the absence of light (darkness, black bar). B, Quantitative analysis of looping in *bel rev* (*N* = 7). Overall, the number of circles per minute differed across conditions, *F*(2,12) = 57.55, *p*<0.0001. Looping was much more pronounced at maximum contrast compared to minimal contrast, *t*(6) = 7.89, *p* = 0.0002, and darkness, *t*(6) = 8.13, *p* = 0.0002. The rate of looping was similar at minimal contrast and in darkness, *t*(6) = 2.13, *p* = 0.0767. ns, non significant. ***, significant at *p*<0.001.

CN in *bel rev* mutants is visual-input dependent and contrast sensitive [5]. As suggested by the concurrence of looping and CN, we next asked if looping is also influenced by visual input and contrast, which would further support a role of vision in this behavior. Thus, we recorded swimming traces of *bel rev* with different projected visual scenes: maximum contrast, minimal contrast, or darkness (Figure 2). Compared to the maximum contrast condition (Figure 2A), looping was remarkably reduced at minimal contrast, and virtually absent when the light was off, implicating visual-input and contrast dependence of looping in *bel rev* (Figure 2B, Table S1). The significant difference between maximum contrast and darkness cannot only be attributed to the generally lower activity of fish in darkness since the recorded swimming traces of those fish that were more active in darkness displayed a pattern distinctive from looping (Figure S1). In addition, when exposed to a 360° uniform, contrastless background with luminance, *bel rev* mutants did not exhibit any looping (Movie S2). As a control, we also examined the swimming behavior of both *bel fwd* and wt under the same maximum contrast condition. In contrast to *bel rev*, they both did not show looping behavior (Table S1). Taken together, these results show that looping is visual input dependent and sensitive to visual contrast.

**Figure 3:**
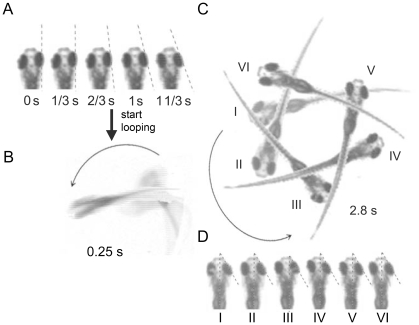
Eye movements before and during looping. A, When presented a stationary stimulus, *bel rev* always showed several instances of congenital nystagmus prior to the onset of looping. B, C, The larva began to loop in the same direction as the preceding eye movements. D, During looping, the eyes stopped moving, assuming a peripheral position toward the looping direction.

Dependence of CN and looping on visual input and contrast raises the question if there is a causal relationship between the afferent input and the efferent response. Close examination of recorded looping (stationary black/white grating; Movie S1) revealed that each occurrence of looping was preceded by several instances of CN, and that the slow phase of CN and subsequent looping had the same direction (Figure 3A and 3B). Furthermore, during looping, the eyes did not move and stayed in a peripheral position oriented towards the looping direction (Figure 3C and 3D). These findings suggest that looping in *bel rev* is triggered, but not maintained, by CN. However, CN is sufficient, but not necessary for the initiation of looping (Movie S4).

### Looping is a compensatory body movement to reduce circular vection caused by retinal slip

Since looping was evoked, but not maintained by CN, we hypothesized that CN may induce looping by virtue of self-motion perception (circular vection). In other words, fish may increasingly perceive the elevating retinal slip generated by CN as a self-motion in the reversed direction. In order to stabilize the body in space, circular vection would then evoke a compensatory response, causing the body to circle in the opposite direction relative to the perceived self-motion. If this hypothesis holds true, looping should also be inducible in wt larva by circular vection. Indeed, a 360°-rotating drum with black/white grating first induced OKR (eye movements tracking the drum, repeatedly reset by saccades), subsequently translating into body movement oriented toward the direction the drum rotated (Movie S3).

Since reversed OKR and CN in *bel rev* are caused by reversal of retinal slip [5], we expected that circular vection induced by projected moving grating would be reversed in *bel rev*, leading to body looping in opposite direction compared to wt. Indeed our experiments confirmed such a reversal of body looping (Movie S4). Unlike with stationary grating, *bel rev* often began to circle without preceding CN, presumably due to sufficient retinal slip created by the moving scene. In conclusion, these results demonstrate that looping in *bel rev* is an attempt to compensate CN-induced illusory circular body motion.

## Discussion

### Body looping is caused by illusory motion perception in achiasmatic zebrafish

In the present study, we illustrated how visual input can manipulate body balance. The abnormal looping behavior observed in *bel rev* can be attributed to visually-induced self-motion perception (circular vection), caused by prolonged large retinal slip velocity maintained through congenital nystagmus (CN). Normally, when a pattern (i.e., contrast>0) is present in the visual field of a wt fish, spontaneous eye or body movement causes a sudden shift of the image on the retina, leading to a perceived retina slip. In order to re-stabilize the image on the retina (i.e., minimize the retinal slip), the eye or the body adjusts its position respectively. However, since higher brain centers of achiasmatic *bel* (*bel rev*) receive a reversed afference input due to the misprojection of the RGCs, the attempted correction of the eye/body movement actually increases the retinal slip. In other words, the reversed afference input disrupts the negative feedback structure of the optokinetic system and visual-postural control, transforming it to a positive feedback loop that reinforces unstable eye (CN) and body (looping) motion. The predominance of either one or the other compensatory response is most likely determined by whether surround or self motion is perceived. Both types of motion perception (body and world motion) are triggered by retinal slip. In achiasmatic *bel*, the attempt of both postural control and the OKR to reduce the retinal slip ironically contributes to maintaining a high retinal slip.

### Circular vection and looping can be induced in wt fish via whole-field motion stimulus

Most people have sat in an IMAX® movie theater and, at times, felt as if their own body was moving. Any large moving visual scene may cause a sensation of tilting or losing balance when standing or sitting. A recent study in healthy human subjects confirmed that the perception of false self-motion can increase postural instability [19]. Similarly, in wt fish, self-motion-perception can induce looping behavior, which is readily evoked by a rotating 360°-pattern, like a fish IMAX® theater. First, fish may have compensatory eye movement because of perceiving the surrounding scene as rotating, a sensation that gradually, or sometimes abruptly, turns into self-motion perception and causes compensatory looping. The transition between these two types of motion perception is a phenomenon humans are often exposed to in everyday life. For example, when looking out the window at a stationary train on the next track, it appears to depart. We perceive the other train as moving (world-motion), before we begin to feel that our own train is moving (self-motion).

### Afference signal of visual system has a direct input on the postural control

**Figure 4:**
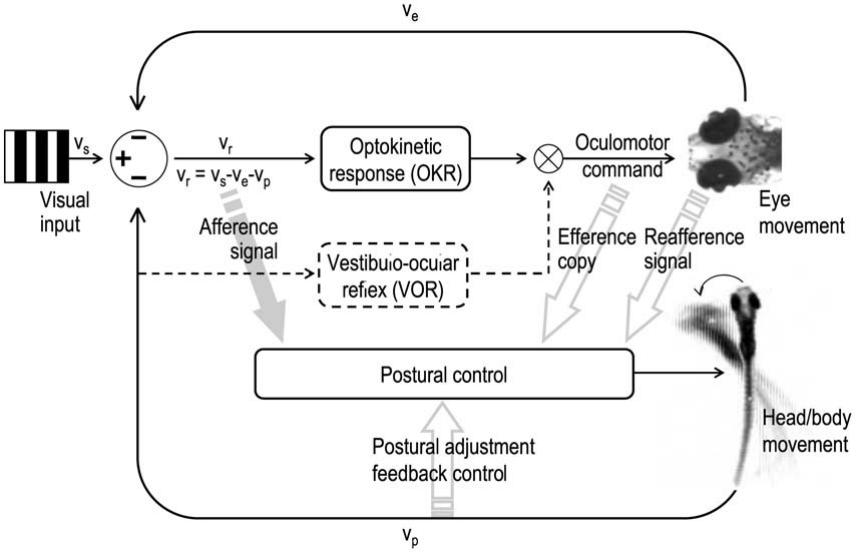
Schematic view of the visual-postural control system. The perceived retinal slip velocity (*v_r_*) is the main afference input signal to the optokinetic response and the postural control system. *v_r_* is physically determined by stimulus velocity (*v_s_*), head/body movement (*v_p_*), and eye velocity (*v_e_*). A non-zero retinal slip velocity serves as the error signal for the optokinetic and the postural feedback loop, evoking either eye movement (Δ*v_e_*) and/or postural adjustment (Δ*v_p_*) in order to compensate the error signal. Eye movements are additionally influenced by the vestibulo-ocular system that triggers eye movement in opposite direction of head/body movement. In addition to the afference signal, the postural control system also receives input from eye movement-related signals (efference copy and reafference signal from the eye muscle). Hence, the stability of the body in space and the visual world on the retina is accomplished through the interplay of both an afference-based feedback system and an eye movement-related feedforward mechanism.

Looping could merely be an outcome of unstable eye movement (CN in this case) via extraocular signals (efference copy and/or reafference signals) as it has been suggested in earlier studies [14]–[16]. However, in this study, we have demonstrated that visual input can directly influence body movement (Figure 2). This indicates that looping may be directly caused by the afference signal. Upon presentation of a stationary grating, eye movement (CN) occurs prior to body movement (looping) with the retinal slip being equivalent to eye movement (see Figure 3A and Movie S1). Once looping starts, the compensatory eye movements cease while looping, leading to a retinal slip that equals to body motion (see Figure 3B–D and Movie S1). Moreover, looping can be initiated without preceding CN when a moving grating is presented (Movie S4). Since in either case no CN occurs during looping, body looping cannot be attributed to any eye-movement-related signal. Rather, we found a constant retinal slip (>0), which together with the contrast requirement strongly implies that the visual-postural control can be directly affected by the retinal slip input (Figure 4).

Earlier reports on similar circus swimming behaviors in tadpoles or other fish date back to the 1940s [20]–[23]. All of these studies manipulated the afference signal (visual input) in such a manner that the retinal slip was reversed: A surgical rotation of the eye ball by 180° [20], collateral transplantation of the eyes [21], or modification of the optic nerve projection to the optic tectum [22], [23] caused similar responses. Interestingly, in addition to looping, some surgically modified fish also showed reversed OKR and spontaneous nystagmus. Based on the present study and our previous report [5], these earlier observations can now be readily explained by the interplay of reversed retinal slip, spontaneous nystagmus, and self- and world-motion perception.

Because of the well developed visual and locomotor system, predatory teleosts are excellent models for studying the relationship between vision and body movement [24]. We used zebrafish as our behavioral model to address this issue. This current study may also shed light on head nodding or tremor, a pathology present in some CN patients [25]–[27], which may serve to compensate vection phenomena induced by instable eye movements in a similar way as looping does in *bel rev*. This together with the strong resemblance between the eye oscillations of *bel rev* and CN in human patients with defective retinotectal projection [5] emphasizes that *bel rev* is a valuable disease model for CN in humans. In contrast to the “efference/reafference-only” hypothesis from other studies which focused on the importance of eye movements in postural balance [14], [16], we have demonstrated that the afference signal (retinal slip) is crucial for maintaining body balance. However, retinal slip or efference copy alone do not provide the postural balance system with sufficient information on self-motion. Hence, both reafferent retinal slip and efference copy are essential for visual-postural control (Figure 4).

## Materials and Methods

### Fish maintenance and breeding

The *bel* (beltv42) mutant line was maintained and bred as described previously [28]. Outcrossed sibling pairs were set up to identify heterozygous carriers. Clutches of these identification crosses as well as crosses of already identified carriers were used for experiments. Embryos were raised at 28°C in E3 medium (5 mM NaCl, 0.17 mM KCl, 0.33 mM CaCl2, and 0.33 mM MgSO4; [29]) and staged according to development in days post fertilization (dpf). Larvae at 3–4 dpf were anesthetized with 3-aminobenzoic acid ethyl esther methane sulfonate (MESAB) to sort the homozygous mutants according to their eye pigmentation phenotype [1].

### Analysis of swimming behavior

A modified version of a previously described optokinetic response setup [30] was used. To study the swimming behavior, larvae aged between 5 to 6 dpf were placed either in a round 16 mm (to allow better visualization of eye movements, see Figure 4) or otherwise in a 22 mm diameter dish filled with water. Using a digital light projector (HP vp6111), moving and still vertical sine-wave gratings (spatial frequency (spf) = 0.06 cycles/deg) were projected onto a cylindrical diffusion screen. The dimensions of the projected image were approximately 99° horizontally and 52° vertically with the screen having a distance of approximately 4.65 cm to the center of the container. The behavior of the fish was recorded by an infrared-sensitive black/white CCD camera (Sony XT-SC50; Berlin, Germany) at a rate of 12.5 frames per second. Subsequently, the frames were transformed to movies with VideoMach 2.7.2 (www.gromada.com). To increase the field of view, the camera was mounted onto a 35 mm lens with a custom built adapter in place of the microscope. Fish were traced with a custom built program, written in the LabView language (LabView IMAQ version 7.1, National Instruments), which used the “match pattern 2” module of IMAQ Vision 3.1 (National Instruments) to localize larvae. Images in which the larvae could not be localized were excluded.

### Experimental design

#### Contrast dependence (projected image)

The swimming behavior of seven *bel rev* was analyzed under three different conditions: Maximum contrast (Max): A still sine-wave grating (spf = 0.06 cycles/deg) with 100% contrast [30] was projected. Minimal contrast (Min): A homogeneous gray image with no contrast was projected onto the diffusion screen with the same light intensity as the averaged intensity under maximum contrast. Darkness (D): The light beam was disrupted with a nontransparent box. The order of presentation was Min-Max-D-Max with each condition lasting one minute. This sequence was repeated three to four times for a single fish (denoted as R in Table S1). To evaluate the looping behavior, completed circles per condition were counted independently by two raters (one blinded). The number of observed circles never differed more than one circle per condition. For each fish under each condition, the mean circles per minute were, first, averaged between raters within each block, and then, averaged across repeated blocks (denoted as M in Table S1). As a control, we analyzed the swimming behavior of six *bel fwd* and six wild-type (wt) larvae under maximum contrast (Max) condition for four minutes.

#### Contrast dependence (360° stimulus)

Due to the restricted size of the projected image (99° horizontally), it was not possible to produce a uniform surrounding with no contrast. The scene outside the projected image would always be darker than the projected image, therefore creating a contrast. In order to create a 360° homogenous visual scene, we fixed a white paper around a dish of 30 mm diameter in size. Similarly, for creating a 360° visual scene with a square grating, black and white bars of equal width (spf approx. 0.06 cycles/deg) were painted on the paper that was wrapped around the dish. To show that *bel rev* show no looping when presented a uniform white background, we analyzed the swimming behavior of the larva in response to a white background (2×3 min) and a high contrast background (2×3 min). The movie was recorded with an Allied Vision Pike camera (Movie S2).

#### Moving grating (projected image)

To test if the looping direction is influenced by the initial velocity of the retinal slip, we projected a grating that moved back and forth (16 s clockwise, 16 s counter-clockwise, etc.) at a constant velocity of 16°/s (spf = 0.06 cycles/deg; contrast = 100%) onto the diffusion screen.

#### Moving grating (360° stimulus)

We used a rotatable drum, fitted with black and white bars, to investigate the behavior of wt and *bel rev* in response to ganzfeld stimuli. Larvae were placed in a round 22 mm diameter dish in the center of the drum (one revolution = 25 s = >14.4°/s; 7 stripes per 360° = >0.02 cycles/deg; center to screen = 2.5 cm).

### Statistical analysis

All statistical tests were performed at a significance level of α = 0.01. Loops per minute (averaged within fish over repeated conditions, see Table S1) obtained in the contrast-dependence experiment were compared across conditions, using a one-way repeated-measures ANOVA. Since the omnibus test was significant (*p*<0.0001), pair-wise comparisons between conditions were conducted using paired t-tests (Figure 2B).

## Supporting Information

Figure S1 Swimming trace of a bel rev larva. A, Looping at maximum contrast. B, Distinctive swimming pattern in complete darkness
(0.24 MB TIF)

Table S1 (Circles with different stimulus contrast) and figure movie legends
(0.06 MB DOC)

Movie S1 Looping of bel rev larva as a response to a projected stationary black/white grating. Congenital nystagmus can be observed in this movie.
(1.06 MB MP4)

Movie S2 Looping of bel rev larva when exposed to 360° uniform, contrastless background with luminance alternating with black/white stationary grating background at 3-minute intervals
(6.08 MB MOV)

Movie S3 Looping of wt larva when exposed to ganzfeld motion. This move was recorded at double speed.
(0.76 MB MOV)

Movie S4 Looping of bel rev larva when presented a projected moving black/white grating. The direction of looping can be readily manipulated by the moving scene. Looping occurs in opposite direction than the moving scene.
(1.46 MB MOV)
