# Supplementary figures and images for: Illusionary Self-Motion Perception in Zebrafish
Source: PLoS One. 2009 Aug 12;4(8):e6550. doi: 10.1371/journal.pone.0006550 (PMC2717804; doi:10.1371/journal.pone.0006550)

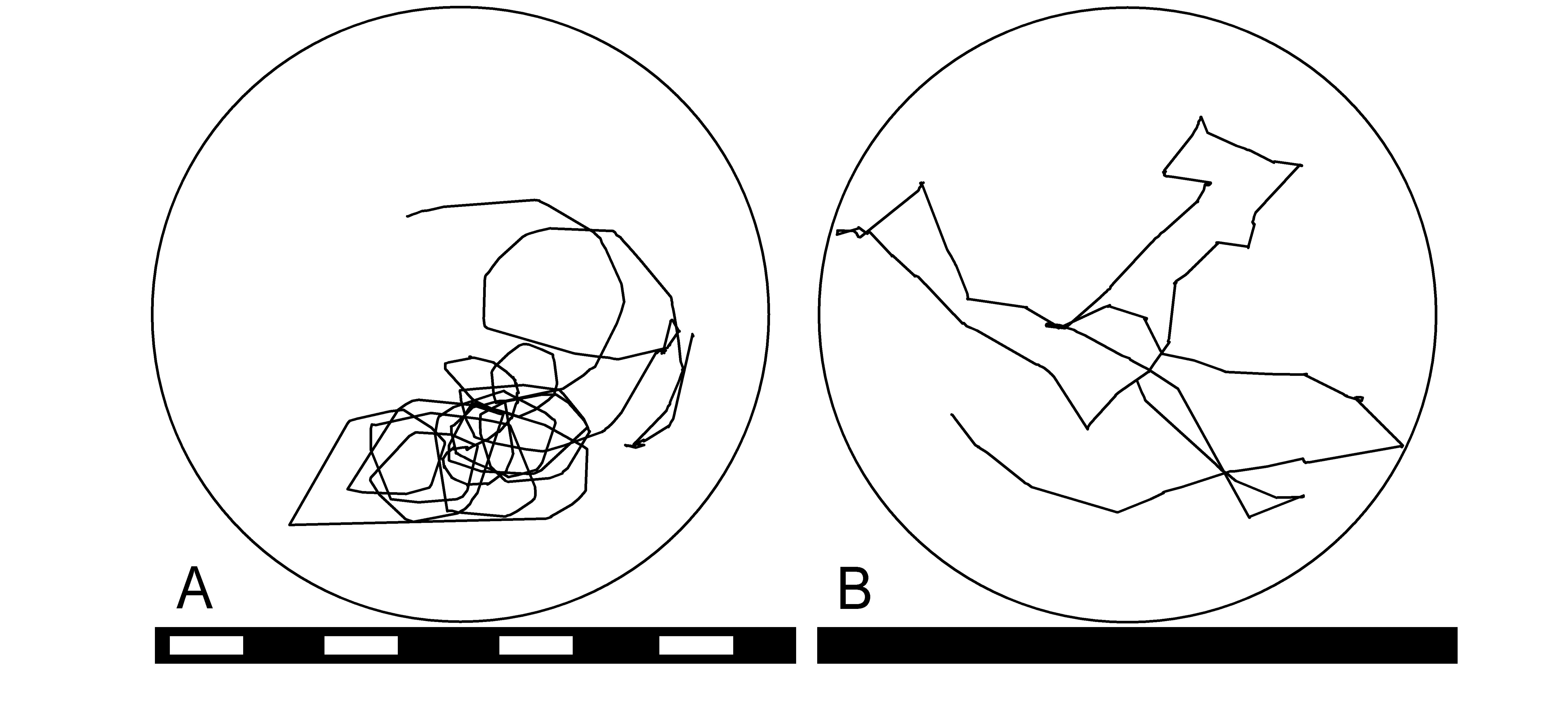

Supplement: Figure S1 — Swimming trace of a bel rev larva. A, Looping at maximum contrast. B, Distinctive swimming pattern in complete darkness (0.24 MB TIF) [file pone.0006550.s001.tif]
