# Supplementary material for: Illusionary Self-Motion Perception in Zebrafish
Source: PLoS One. 2009 Aug 12;4(8):e6550. doi: 10.1371/journal.pone.0006550 (PMC2717804; doi:10.1371/journal.pone.0006550)
Supplement: Table S1 — (Circles with different stimulus contrast) and figure movie legends (0.06 MB DOC) [file pone.0006550.s002.doc]

# Supporting Information

**Table S1.** Circles per minute with different stimulus contrast

|  | Minimum contrast | | |  | Darkness | | |  | Maximum contrast | | |  | Maximum contrast | | |  | Maximum contrast | | |
| --- | --- | --- | --- | --- | --- | --- | --- | --- | --- | --- | --- | --- | --- | --- | --- | --- | --- | --- | --- |
| Fish | *M* | *SEM* | *R* |  | *M* | *SEM* | *R* |  | *M* | *SEM* | *R* | Fish | *M* | *SEM* | *R* | Fish | *M* | *SEM* | *R* |
| 1 | 1.67 | 1.20 | 3 |  | 0.00 | 0.00 | 3 |  | 4.42 | 1.43 | 6 | 8 | 0.00 | 0.00 | 4 | 14 | 0.00 | 0.00 | 4 |
| 2 | 1.50 | 1.02 | 4 |  | 0.00 | 0.00 | 4 |  | 6.00 | 1.21 | 8 | 9 | 0.00 | 0.00 | 4 | 15 | 0.00 | 0.00 | 4 |
| 3 | 0.38 | 0.24 | 4 |  | 0.00 | 0.00 | 4 |  | 6.81 | 1.61 | 8 | 10 | 0.00 | 0.00 | 4 | 16 | 0.00 | 0.00 | 4 |
| 4 | 0.75 | 0.25 | 4 |  | 0.00 | 0.00 | 4 |  | 5.50 | 1.03 | 8 | 11 | 0.00 | 0.00 | 4 | 17 | 0.00 | 0.00 | 4 |
| 5 | 0.13 | 0.13 | 4 |  | 0.25 | 0.25 | 4 |  | 4.44 | 1.19 | 8 | 12 | 0.00 | 0.00 | 4 | 18 | 0.00 | 0.00 | 4 |
| 6 | 0.00 | 0.00 | 4 |  | 0.00 | 0.00 | 4 |  | 2.31 | 0.66 | 8 | 13 | 0.00 | 0.00 | 4 | 19 | 0.00 | 0.00 | 4 |
| 7 | 0.00 | 0.00 | 4 |  | 0.00 | 0.00 | 4 |  | 3.63 | 1.16 | 8 |  |  |  |  |  |  |  |  |
| Avg. | 0.63 | 0.27 | N=7 |  | 0.04 | 0.04 | N=7 |  | 4.73 | 0.57 | N=7 | Avg. | 0.00 | 0.00 | N=6 | Avg. | 0.00 | 0.00 | N=6 |

Note: R, number of repeated blocks. M, mean circles per minute averaged across blocks. SEM, standard error of the mean. Fish 1 to 7 were *bel rev*, 8-13 were *bel fwd*, 14-19 were wt.

**Figure S1.** Swimming trace of a *bel rev* larva. A, Looping at maximum contrast. B, Distinctive swimming pattern in complete darkness.

**Movie S1.** Looping of *bel rev* larva as a response to a projected stationary black/white grating. Congenital nystagmus can be observed in this movie.

**Movie S2.** Looping of *bel rev* larva when exposed to 360° uniform, contrastless background with luminance alternating with black/white stationary grating background at 3-minute intervals.

**Movie S3.** Looping of wt larva when exposed to ganzfeld motion. This movie was recorded at double speed.

**Movie S4.** Looping of *bel rev* larva when presented a projected moving black/white grating. The direction of looping can be readily manipulated by the moving scene. Looping occurs in opposite direction than the moving scene.
